# Next-generation Sequencing and Other Second Tier Tests in Newborn Screening for (X-linked) Agammaglobulinemia

**DOI:** 10.1007/s10875-025-01927-6

**Published:** 2025-11-08

**Authors:** Maartje Blom, Annelotte J. Duintjer, Ingrid Pico-Knijnenburg, Sandra Imholz, Sahila Balkassmi, Hermine A. van Duyvenvoorde, Mirjam van der Burg

**Affiliations:** 1 Department of Pediatrics, Laboratory for Pediatric Immunology, Willem- Alexander Children’s Hospital, Leiden University Medical Center (LUMC), Albinusdreef 2, Leiden, 2333 ZA the Netherlands; 2 Centre for Health Protection, National Institute for Public Health and the Environment, Bilthoven, the Netherlands; 3 Clinical Genetics, Leiden University Medical Center (LUMC), Leiden, the Netherlands

**Keywords:** X-linked agammaglobulinemia, XLA, Bruton tyrosine kinase, BTK, B-cell deficiency, Newborn screening, NBS, Kappa-deleting recombination excision circles, KREC, Genetics, Next generation sequencing, NGS

## Abstract

**Purpose:**

Patients with X-linked agammaglobulinemia (XLA) suffer from severe, recurrent infections potentially leading to life-threatening complications. Early diagnosis and timely treatment can prevent infections and secondary complications, emphasizing a role for newborn screening (NBS). NBS for XLA is based on quantification of kappa-deleting recombination excision circles (KRECs). KREC-based screening could result in a large number of false-positive referrals associated with high impact for parents and health care systems, indicating the need for a second tier test.

**Methods:**

KRECs were measured in NBS cards (*N* = 110,491) with a multiplex TREC/KREC qPCR assay. As second tier test options, an alternative qPCR multiplex assay, epigenetic immune cell counting for relative B-cell quantification and targeted next-generation sequencing with B-cell deficiency gene panel including 73 genes were performed on NBS cards of newborns with low KRECs.

**Results:**

In total, 136/110,491 newborns had KRECs below cut-off. With the alternative qPCR multiplex assay, 16/110 of these newborns (14.5%) had KRECs above cut-off and would not have been referred. With epigenetic immune cell counting, 16.5% (17/103) had relative B-cell counts in the range of healthy controls. Targeted NGS showed promising results as 87 out of 103 (84%) newborns with low KRECs did not show any pathogenic/likely pathogenic variants and would not have been referred for follow-up diagnostics.

**Conclusion:**

Several second tier tests can potentially reduce the number of false-positive referrals in NBS for XLA. NGS seems to be the most effective technique in NBS for XLA and other forms of agammaglobulinemia. Our results show promising first steps towards the implementation of NBS for XLA.

**Supplementary Information:**

The online version contains supplementary material available at 10.1007/s10875-025-01927-6.

## Introduction

X-linked agammaglobulinemia (XLA) is an inborn error of immunity (IEI) caused by pathogenic variants in the *Bruton’s tyrosine kinase* (*BTK*) gene located on the long arm of the X chromosome [1–3]. BTK is a signal-transducing protein and pathogenic variants in the *BTK* gene cause a block in the differentiation of B-lymphocyte progenitors leading to severely decreased levels of all serum immunoglobulins [4]. Autosomal recessive (AR) and autosomal dominant (AD) forms of agammaglobulinemia and other genetic defects affecting early B-lymphocyte development have been reported as well [3, 5].

Patients with XLA and other forms of agammaglobulinemia are highly susceptible to recurrent infections with encapsulated bacteria, most frequently resulting in upper and lower respiratory tract infections [4, 6, 7]. Chronic diarrhoea caused by *Giardia lamblia* and susceptibility to enteroviruses leading to meningoencephalitis are described as well [4, 6, 7]. Treatment consists of life-long administration of immunoglobulins either intravenously or subcutaneously. In addition, prophylactic antibiotics, careful monitoring and supportive therapies play a role in the treatment of XLA patients [8, 9]. Without treatment, agammaglobulinemia may lead to chronic lung disease (CLD) and irreversible lung damage, which is the main cause of mortality [10–12].

Early detection and timely start of immunoglobulin replacement therapy (IGRT) is suggested to reduce infections and non-infectious complications in patients with B-lymphocyte deficiencies [10, 12]. Recent studies have shown that XLA patients with an early diagnosis were less likely to develop lower respiratory tract infections [13–15]. As repeated episodes of these types of infections are highly associated with the development of CLD and bronchiectasis, early diagnosis could potentially reduce morbidity and mortality in these patients. Research additionally shows that after diagnosis and initiation of IGRT, rates of severe invasive infections such as sepsis and meningitis declined [16]. Nevertheless, despite appropriate IGRT and supportive therapies, patients are not infection free and continue to experience complications suggesting the need for alternative treatment options [14–16].

Early detection of B-lymphocyte deficiencies can be realized by newborn screening (NBS) with the detection of kappa-deleting recombination excision circles (KRECs) in dried blood spots (DBS). KRECs are circular, episomal DNA elements formed during V(D)J recombination of the *IGK* locus during B-cell maturation [17]. As these circular elements persist in the cell and are unable to replicate, they are considered to be indirect markers of bone marrow output of B-lymphocytes [18]. Previous studies have shown that XLA and other forms of agammaglobulinemia characterized by an absence of B-lymphocytes in the peripheral blood, can be identified by low or absent KRECs measurements with qPCR [19, 20].

KRECs can be measured simultaneously with T-cell receptor excision circles (TRECs) allowing NBS for both B- and T-cell deficiencies such as severe combined immunodeficiency (SCID) at relatively low cost. Some countries have already implemented the TREC/KREC multiplex assay in their NBS program or have performed pilot studies for detection of SCID and XLA [21–30]. These studies have shown successful identification of B-lymphocyte deficient patients shortly after birth, but additionally showed relatively high numbers of newborns with low KREC levels due to differing underlying causes. Low KREC levels can be observed in premature infants as B-lymphopenia can be attributed to an immature development of the adaptive immune system [31]. In addition, patients with congenital disorders such as Jacobsen syndrome, Wiskott–Aldrich syndrome, Dyskeratosis Congenita or newborns of mothers using immunosuppressant medication during pregnancy can present with low B-cells at birth [21, 31–33]. Secondary findings seem inevitable when using KREC analysis as first tier test in a NBS program, but high numbers of (false positive) referrals can lead to high workload for downstream referral centres and excessive diagnostics costs. In addition, (false positive) referrals are associated with high emotional impact for parents and NBS programs should aim to limit the harm-benefit ratio of the screening program [34]. For these reasons, many countries are debating whether NBS for agammaglobulinemia will exceed the harm/benefit ratio of a screening program. A second tier test applied after initial KREC measurements could potentially reduce the number of false positive referrals, overcoming the hurdles associated with high referral rates and increasing the positive predictive value of the NBS program. The aim of this study is therefore to examine potential suitable techniques that could be carried out as a second tier test after KREC-analysis in the detection of XLA and other forms of agammaglobulinemia.

## Methods

### Study Population

From April 2018 to December 2019 anonymized heel prick cards (*N* = 110,491) from the Dutch NBS program were included for multiplex TREC/KREC analysis. If parents objected to the use of their newborn’s heel prick card for anonymized research purposes, NBS cards were omitted for KREC analysis. Samples with both low TRECs and KRECs were excluded from second tier analysis, as these newborns were referred to a paediatric-immunologist within the national SCID screening program. Some NBS cards were excluded for second tier analysis due to insufficient DBS material. The use of NBS cards was approved by the National Institute for Public Health and the Environment (RIVM; no 2019–3).

Variables such as gender, gestational age, birth weight, age at sample collection and receiving a blood transfusion less than 24 h prior to sample collection were collected from the Dutch Praeventis NBS database (National Institute for Public Health and the Environment, Bilthoven, the Netherlands).

NBS cards of healthy newborns (*N* = 80) and newborns with low KREC levels (*N* = 110) were analysed with a second TREC/KREC assay. Relative quantification of B-cell counts was performed in NBS cards of healthy controls (*N* = 311) and newborns with low KRECs (*N* = 103) with epigenetic immune cell counting. A total of 103 newborns with low KRECs were included for next generation sequencing (NGS). Original NBS cards of confirmed XLA patients with a known genetic defect were included as positive controls (*N* = 4).

### KREC Measurements and Epigenetic Immune Cell Counting

Initial KREC measurements were performed with the SPOT-it™ Neonatal Screening kit (ImmunoIVD, Stockholm, Sweden) according to manufacturer’s instructions. If KREC levels were below cut-off value (≤ 6 copies/3.2 mm punch), analysis on the same DBS card were repeated in duplicate. As a second tier option, KREC levels were measured with an alternative multiplex qPCR assay, the NeoMDx TREC/KREC/SMN1 multiplex assay (PerkinElmer/Revvity, Turku, Finland) according to the manufacturer’s instructions. RRP30 was used as an internal control. Epigenetic immune cell counting was performed by amplification of cell-type-specific demethylated genomic regions according to the protocol of the manufacturer (Epimune GmbH, Berlin, Germany). Relative (epi) B-cell counts (% of BLC demethylated copies of GAPDH demethylated copies) were calculated as previously described [35]. For more details on the material and methods see [36].

### Next Generating Sequencing Panel and Workflow

An IonAmpliSeq™ customized B-cell deficiency panel was developed based on the classification of the International Union of Immunological Societies (IUIS) [5]. Genes were included in the panel if variants in that gene may result in the immunological phenotype of having reduced B-cells at birth. A total of 73 genes were included that met this criterium.

DNA was extracted from one 3.2 mm DBS punch using a lysis-based method from Thermo Fisher Scientific™. Libraries with two primer pools were prepared using the Ion AmpliSeq™ Library Kit with an input of 7.5 ng DNA per sample. Two amplification cycles were added to the number of cycles as described in the protocol to amplify target regions, since DNA input was lower than recommended by the protocol. A customized workflow for alignment and germline variant calling was carried out in Ion Reporter ™ 5.20.2.0. Sequence reads were aligned to the reference genome hg19.

### Variant Filtering and Interpretation

Variants that passed the following criteria were included for further analysis and interpretation: 1) located in an exon or at splice sites [2], minor allele frequency (MAF) ≤ 0.02 [3], allele frequency in the European non-Finnish population ≤ 0.02 (Genome Aggregation Database (gnomAD) ExAC ENFAF) and [5] a prevalence of the variant of ≤ 5 in all sequenced samples. Synonymous variants or variants with unknown effect were excluded from further analysis [6].

Furthermore, variants were only further analysed if they met adequate quality control (QC) parameters. Phred quality scores and coverage were taken into account to evaluate overall variant QC. Variants were excluded from further analysis if they had one of the following QC parameters: (1) a phred quality score < 20 and a coverage < 15, or (2) a coverage > 17 with a phred quality score < 14, or (3) a phred quality score > 20 but a coverage < 9.

Variant annotation was conducted using Alamut™ Visual Plus (version 1.9). GnomAD was consulted to obtain population frequency data. REVEL scores and CADD scores were evaluated as in-silico pathogenicity predictors. Furthermore, classification of variants according to the American College of Medical Genetics and Genomic (ACMG) guidelines was assessed. Finally, ClinVar and the Leiden Open Variation Database (LOVD) were consulted as variant databases. Variants were divided into three categories: (likely) benign (LB/B), variant of uncertain significance (VUS) and (likely) pathogenic (LP/P).

### Statistical Analysis

Descriptive statistics were used to summarize the distribution of KREC levels and relative (epi) B- counts. For correlation analysis, Pearson r correlation tests were used, while unpaired t-tests were used for group comparison. Epigenetic BLC/GAPDH copies were log-transformed and used to estimate a normal distribution with 99.9% confidence interval. P-values < 0.05 were considered statistically significant. All P-values are two-sided. Statistical analysis was carried out with SPSS version 29.0 for Windows (SPSS, Inc., Chicago, IL, USA).

## Results

### Characteristics of the Study Population

From April 2018 to December 2019 anonymized heel prick cards (*N* = 110,491) from the Dutch NBS program were included for multiplex TREC/KREC analysis. After repeated analysis in triplicate, 136 newborns had KREC levels below the cut-off value of ≤ 6 copies/3.2 mm punch. The distribution of KREC levels in the population is depicted in Figure S1. The characteristics of these newborns are presented in Table 1. Six samples had both TREC- and KREC levels below the cut-off value and were excluded for second tier analysis as these newborns were referred within the SCID screening program. Two samples had β-actine levels below ≤ 1000 copies/3.2 mm punch and would require an immediate second DBS sample in a screening program. There were 32 preterm newborns (defined as < 36 weeks of gestational age) with low KREC levels that would get a second heel prick at the corrected term gestational age (after ≥ 36 weeks), resulting in 96 newborns that would potentially be directly referred to a paediatric-immunologist based on KREC measurements only.

**Table 1:** Characteristics of the newborns with low KREC levels (*N* = 136) defined as krecs ≤ 6 copies/3.2 mm punch. Certain text was bold in this Table to highlight the headers

Parameters	Total *N* = 136
**Median KREC values (copies/3.2 mm punch; IQR)**	2.9 (1.40–4.30)
**Median β-actin (copies/3.2 mm punch; IQR)**	2476 (1476–3740)
**Sex % (** *N* **)**	
Male % (*N*)	58.1% (*N* = 79)
Female % (*N*)	41.9% (*N* = 57)
**Age at sample collection (in hours), median (IQR)**	90 (83–105)
**Gestational age (in days), median (IQR)**	272 (249–283)
Extremely preterm < 32 weeks % (*N*)	13.2% (*N* = 18)
Preterm (32–36 weeks) % (*N*)	10.3% (*N* = 14)
Term ≥ 36 weeks % (*N*)	76.5% (*N* = 104)
**Birth weight (in grams), mean SD**	2908 (SD 26.5)
Low birth weight < 2500 gram % (*N*)	30.1% (*N* = 41)
Normal birth weight ≥ 2500 gram % (*N*)	69.9% (*N* = 95)
**Blood transfusion < 24 h sample collection**	
Yes % (*N*)	1.5% (*N* = 2)
No % (*N*)	98.5% (*N* = 134)

*IQR = interquartile range, SD = standard deviation

### KREC Measurements with an Alternative Multiplex qPCR Assay as Second Tier Test

**Fig. 1:**
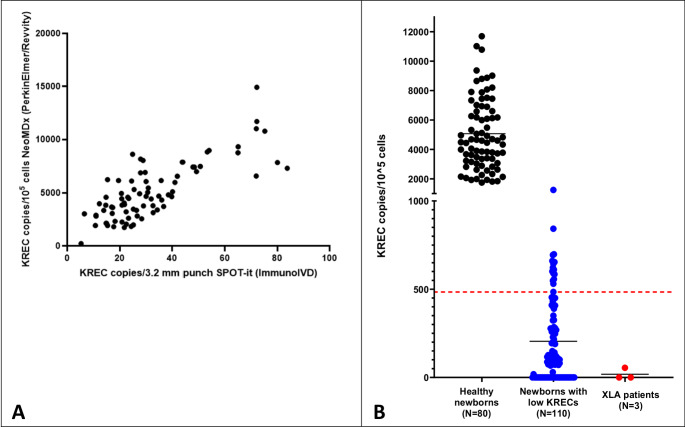
KREC copies analysed with a second qPCR assay. **A. **Correlation plot of KREC copies analysed with both assays (SPOT-it, ImmunoIVD and NeoMDx, PerkinElmer/Revvity) in healthy controls (*N* = 80), Pearson R correlation was 0.83 (*P* < 0.01). **B. **KREC levels in copies/10^5^ cells of healthy newborns (*N* = 80), newborns with low KREC levels (*N* = 110) and confirmed XLA patients (*N* = 3) measured with the NeoMDx assay (PerkinElmer/Revvity). The red dotted line is the cutoff of the manufacturer at KREC ≤ 459 copies/10^5^ cells. The black line shows mean KREC copies/10^5^ cells

The commercially available NeoMDx qPCR was used as a second tier to examine whether presence of single-nucleotide polymorphisms (SNPs) in the KREC targeted region could potentially lead to false-positive referrals. The mean KREC value of healthy controls (*N* = 80) was 5129 copies/10^5^ cells (SD 2641 copies/10^5^ cells; range 1744–14917 copies/10^5^ cells), whereas the mean KREC value of the low KREC samples (*N* = 110) was 204.8 copies/10^5^ cells (SD 22.82 copies/10^5^ cells; range 0-1244 copies/10^5^ cells). Pearson r correlation between KREC levels measured with the SPOT-it kit assay and the NeoMDx assay was 0.83 (*P* < 0.01) (Fig. 1A). Sixteen out of 110 newborns (14.5%) with low KREC levels measured with the SPOT-it assay had KREC levels above the cut-off value proposed by the manufacturer (≤ 459 copies/10 cells). All three confirmed XLA patients had KREC levels below the cut-off value (Fig. 1B).

### Epigenetic Immune Cell Counting as a Second Tier Test

**Fig. 2:**
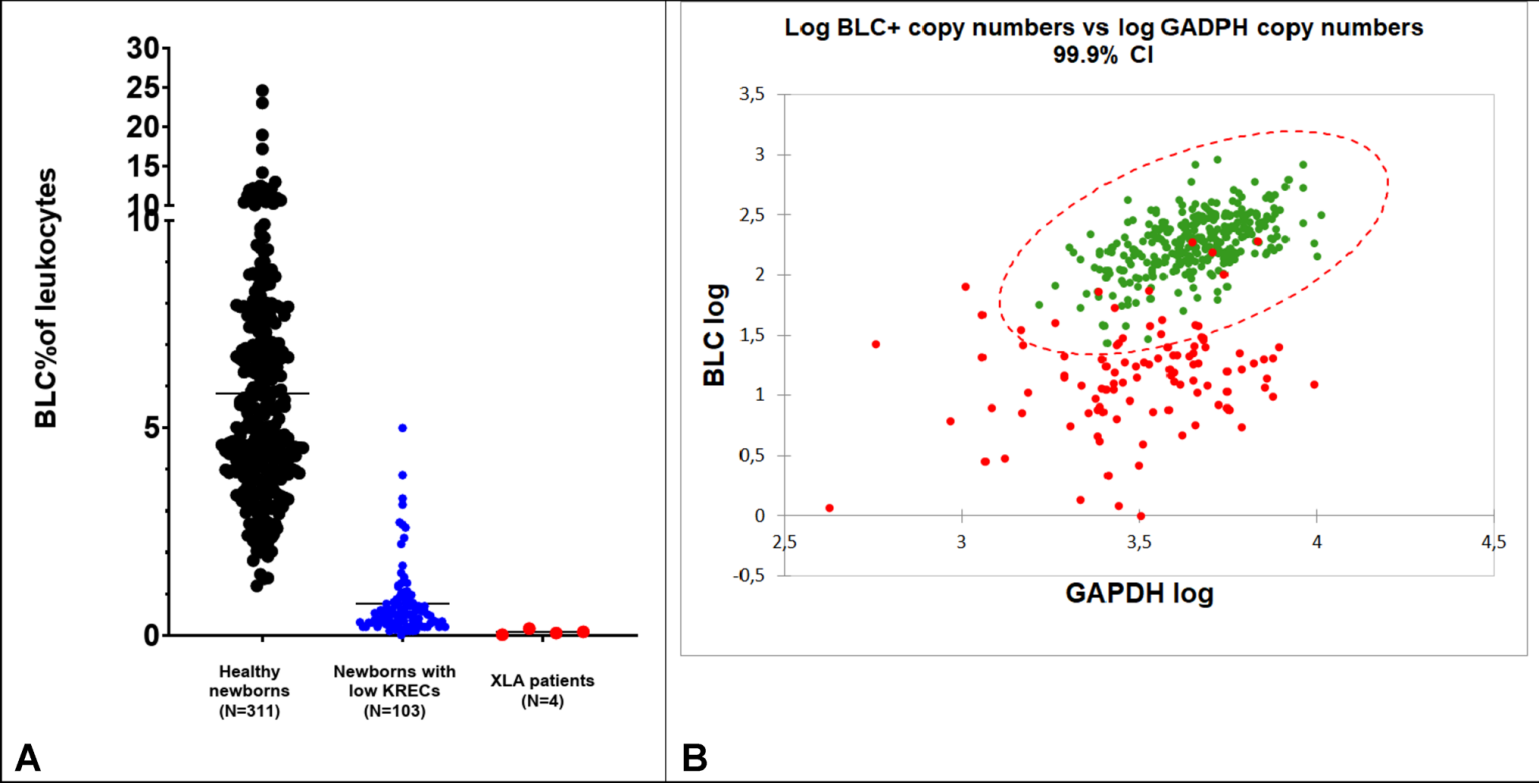
Epigenetic immune cell counting of B lymphocytes. **A. **Relative (epi) BLC counts as a percentage of total leukocytes of healthy newborns (*N* = 311), newborns with low KRECs (*N* = 103) and XLA patients (*N* = 4) measured with epigenetic qPCR (Epimune GmbH). The mean is depicted with a black line. **B. **Log-transformed epigenetic BLC/GAPDH copies with a 99.9% confidence interval (red ellipse). Healthy newborns (*N* = 311) are depicted in red and newborns with low KREC levels are depicted in green (*N* = 103)

Epigenetic immune cell counting is based on amplification of a B-cell-specific demethylated genomic region and measurement of relative (epi) B-cell counts in DBS. Mean relative (epi) B-cell count as a percentage of leukocytes (BLC%) in healthy newborns was 5.84% (*N* = 311; range 1.2–24.6%), while mean relative (epi) BLC count for newborns with low KRECs was 0.76% (*N* = 103; range 0–5.0%) (*P* < 0.001) Pearson r correlation between KREC and unmethylated BLC copies was 0.73 (*P* < 0.001). Fifteen of 103 newborns with low KRECs had relative (epi) B-cell counts in the range of healthy controls (14.6%; Fig. 2A). For (epi) B-cell counts and GAPDH measurements, 17 out of 103 newborns with low KREC levels (16.5%) were within the 99.9% confidence interval ellipse of the healthy controls (Fig. 2B).

### Next Generation Sequencing (NGS) as a Second Tier Test

A customized B-cell deficiency panel with 73 genes in which variants may result in reduced B-cells at birth was used for targeted amplicon sequencing as a second tier test (Supplemental File 1). In total, 103 NBS cards of newborns with low KRECs and NBS cards of 4 confirmed XLA patients were included. The average DNA concentration after extraction was 1 ng/uL. After applying the filtering strategy, three low KREC samples had no identified filtered variants in the 73 genes analysed. Therefore, a total of 503 filtered variants were detected distributed over 100 low KREC samples and 4 XLA samples. Genes in which variants were identified are included in Figure S2. Within all filtered variants, 84 (17%) variants distributed over 29 (27%) samples, did not meet QC criteria and were excluded, resulting in 419 variants for further analysis.

### Scoring System Sequence Artefacts

**Fig. 3:**
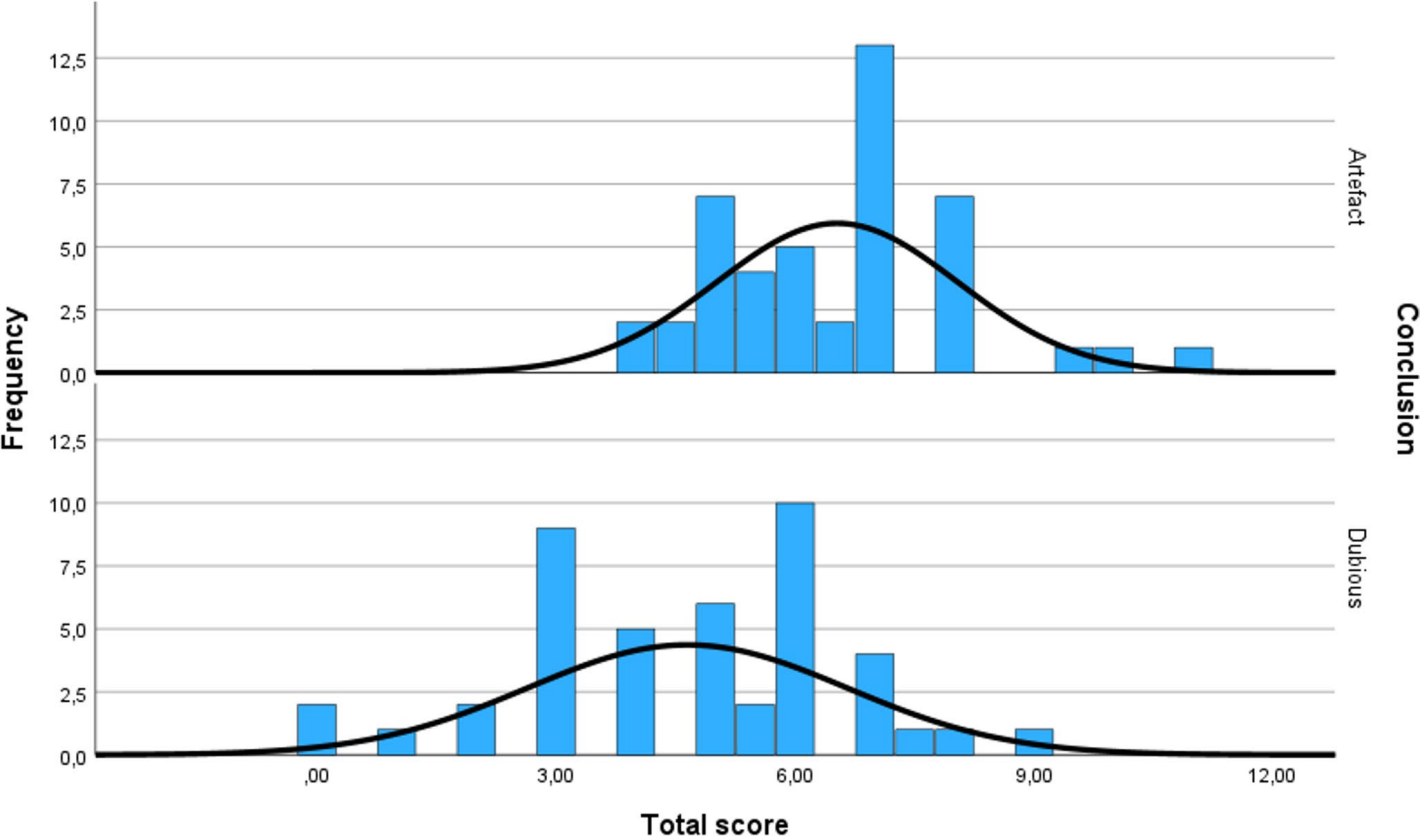
Scores of evident artefacts compared to suspected indel variants

A scoring system was developed to help distinguishing sequence artefacts from actual variants within a NBS setting to improve inter-rater reliability and consistency, with a higher score indicating more likeliness of the variant being an artefact (Table S1). The scoring system was applied to small indels suspected of being the result of sequence artefacts. Several characteristics that are indicative for sequence artefacts were included. First of all and most importantly, scores were given in case small indels were called in a homopolymer stretch as it is known that homopolymer errors are common when using the Ion Torrent platform [37–39]. Furthermore, higher scores were assigned when these indels were present in multiple samples. Points were also given for variants with high susceptibility of strand bias, meaning alternative bases were highly represented in only one strand, thus either in the forward strand or either in the reverse strand [40, 41]. Moreover, points were given to heterozygous variants with an alternative allele ratio < 0.25 [42]. Points were taken in case a variant was described in population databases. Finally, phred quality scores and coverage were also taken into account in scoring. To determine a threshold, evident sequence artefacts were scored to compare outcomes. Based on this comparison, the evident sequence artefacts scored higher than the suspected indels with means (SD) of respectively 6.53 (1.51) as compared to 4.65 (2.00). Evident artefact scores ranged from 4 to 11 with a median score of 7 and scores of suspected indels ranged from 0 to 8.75 with a median of 5. Evident artefacts scored not lower than 3, indicating that suspected indels scoring lower than 3 were less likely to be the result of a sequence artefact and therefore require critical assessment and interpretation. Suspected indels that scored higher than 3 were assumed to be the result of a sequence artefact and were thus classified as being so (Fig. 3).

### Variant Interpretation in Next Generation Sequencing

From 419 filtered variants that passed QC, 135 (32%) variants were classified as LB/B, 119 (28%) variants were classified as a VUS and 74 (18%) base alterations were called as variant but were actually sequence artefacts. A total of 70 (17%) variants concerned heterozygous LP variants in an AR gene with no second VUS/(L)P variant in that gene and did therefore not led to further consequences. Finally, 21 (5%) variants in AD genes (or 2 variants in AR genes) classified as LP/P and were distributed over a total of 19 samples. This includes the LP/P variants detected in the *BTK* gene within the 4 XLA patients (Table 2). Thus, LP/P variants were detected in a total of 15 low KREC samples (Table 2). Besides the XLA patients, there was also a variant detected in the *BTK* gene in sample low KREC 77. The *BTK* variants in sample low KREC 77 and XLA 1 were also described in the LOVD database.

Furthermore, in two samples there were two variants identified in the same gene, which would, in light of possibility of compound heterozygosity, be an indication for referral for further diagnostic analysis (Table 2). Sample low KREC 74 had two variants classified as a VUS in the *FAT4* gene. Sample Low KREC 101 had one (likely) pathogenic variant and one variant classified as VUS in the *PGM3* gene.

**Fig. 4:**
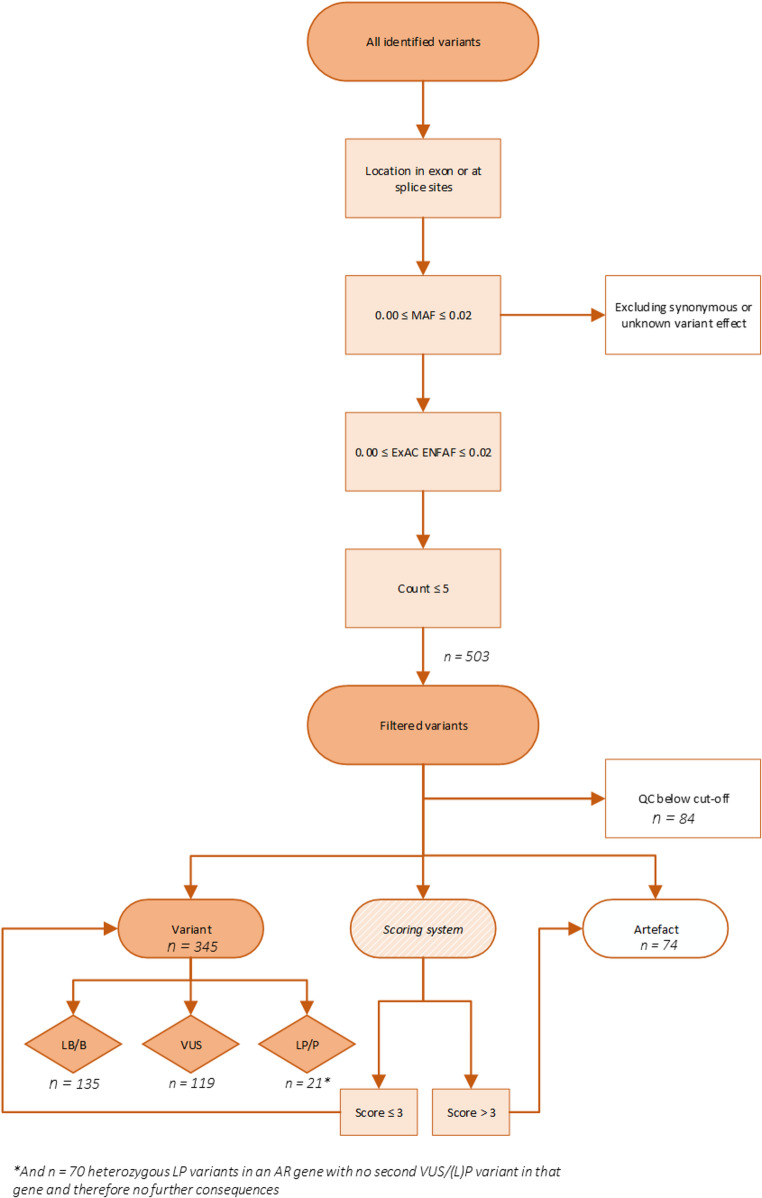
Overview of variant filtering and interpretation of the workflow of NGS as a second tier in NBS for B-cell deficiencies

The overall variant filtering and interpretation workflow is presented in Fig. 4. Based on these results, it would be indicated to refer 16 children with low KRECs to a paediatric-immunologist for further diagnostics.

**Table 2:** Pathogenic variants, likely pathogenic variants and potential heterozygous compound mutations identified in newborns with low KRECs (*N* = 103) and confirmed XLA patients (*N* = 4) with NGS (*AD = autosomal dominant*,* AR = autosomal recessive*,* LOF = loss of function*,* GOF = gain of function*)

Sample ID	Variant	Gene (Inheritance)	GnomAD MAF	ACMG Classification	REVEL score	CADD score^a^	Interpretation
XLA patient 1	NM_000061.3: c.72 C > G p.(Asn24Lys) *Hemizygous*	*BTK* (X-linked)	Not described	VUS (PM1, PM2, PP3)	0.815	24	Pathogenic
XLA patient 2	NM_000061.3: c.1573 C > T p.(Arg525*) *Hemizygous*	*BTK* (X-linked)	Not described	Pathogenic (PVS1, PM2, PP5)	-	-	Pathogenic
XLA patient 3	NM_000061.3: c.919 A > G p.(Arg307Gly) *Hemizygous*	B*TK* (X-linked)	Not described	Likely pathogenic (PM1, PM2, PP3, PP5)	0.909	26.8	Pathogenic
XLA patient 4	NM_000061.3: c.1558 C > T p.(Arg520*) *Hemizygous*	*BTK* (X-linked)	Not described	Pathogenic (PVS1, PM2, PP5)	-	-	Pathogenic
LOW KREC 77	NM_000061.3: c.1837G > A p.(Gly613Ser) *Hemi- or homozygous*	*BTK* (X-linked)	Not described	VUS (PM1, PM2, PP3)	0.897	26.5	(Likely) pathogenic
LOW KREC 7	NM_032415.7: c.1484delC p.(Pro495Argfs*6) *Heterozygous*	*CARD11* (AD LOF)	Not described	Pathogenic (PVS1, PM2)	-	-	(Likely) pathogenic
LOW KREC 24 LOW KREC 95	NM_001068.3: c.1249_1250insT p.(Ala417Valfs*4) *Heterozygous*	*TOP2B* (AD)	Not described	Pathogenic (PVS1, PM2)	-	-	(Likely) pathogenic
LOW KREC 48 LOW KREC 50 LOW KREC 63	NM_182972.2: c.206delG p.(Gly69Valfs*85) *Heterozygous*	*IRF2BP2* (AD)	Not described	Pathogenic (PVS1, PM2)	-	-	(Likely) pathogenic
LOW KREC 65	NM_003998.4: c.1846G > T p.(Gly616Cys) *Heterozygous*	*NFKB1* (AD)	0.0000003977	VUS (PM1, PM2, PP3)	0.721	29.3	(Likely) pathogenic
LOW KREC 67	NM_001136139.4: c.956delG p.(Gly319Alafs*r75) *Heterozygous*	*TCF3* (AD)	Not described	Pathogenic (PVS1, PM2)	-	-	(Likely) pathogenic
LOW KREC 73	NM_001068.3: c.4422delT p.(Phe1474Leufs*3) *Heterozygous*	*TOP2B* (AD)	Not described	Pathogenic (PVS1, PM2)	-	-	(Likely) pathogenic
LOW KREC 76	NM_152703.5: c.4551delA p.(Val1518Serfs*8) *Heterozygous*	*SAMD9L* (AD GOF)	Not described	VUS (PM2)	-	-	(Likely) pathogenic
LOW KREC 85	NM_001136139.4: c.611_612insC p.(Lys204Asnfs*50) *Heterozygous*	*TCF3* (AD)	Not described	Pathogenic (PVS1, PM2)	-	-	(Likely) pathogenic
LOW KREC 91	NM_032415.7: c.1484delC p.(Pro495Argfs*6) *Heterozygous*	*CARD11* (AD LOF)	Not described	Pathogenic (PVS1, PM2)	-	-	(Likely) pathogenic
LOW KREC 104	NM_001077494.3: c.1416_1417delGC p.(Leu473Alafs*32) *Heterozygous*	*NFKB2* (AD)	0.001290	Pathogenic (PVS1, PM2)	-	-	(Likely) pathogenic
XLA 1	NM_012452.3: c.204_205insA p.(Leu69Thrfs*12) *Heterozygous*	*TNFRSF13B* (AD/AR)	0.0004002	Likely pathogenic (PVS1)	-	-	(Likely) pathogenic
LOW KREC 74	NM_024582.5: c.8015 A > T p.(Asp2672Val) *Heterozygous*	*FAT4* (AR)	0.001362	VUS (PM1, PM2, PP3, BP4)	0.396	25	Potential compound heterozygous
	NM_024582.5: c.8947T > G p.(Phe2983Val) *Heterozygous*	*FAT4* (AR)	0.00003184	VUS (PM1, PM2, PP3)	0.696	25.3	
LOW KREC 101	NM_001199917.2: c.698dupA p.(Tyr233*) *Heterozygous*	*PGM3* (AR)	Not described	Pathogenic (PVS1, PM2)	-	-	Potential compound heterozygous
	NM_001199917.2: c.47 A > G p.(Gln16Arg) *Heterozygous*	*PGM3* (AR)	0.00002485	VUS (PM2, BP4)	0.036	0.016	

^a^ Phred Scaled

### Potential Referrals after Second Tier Test Options

**Fig. 5:**
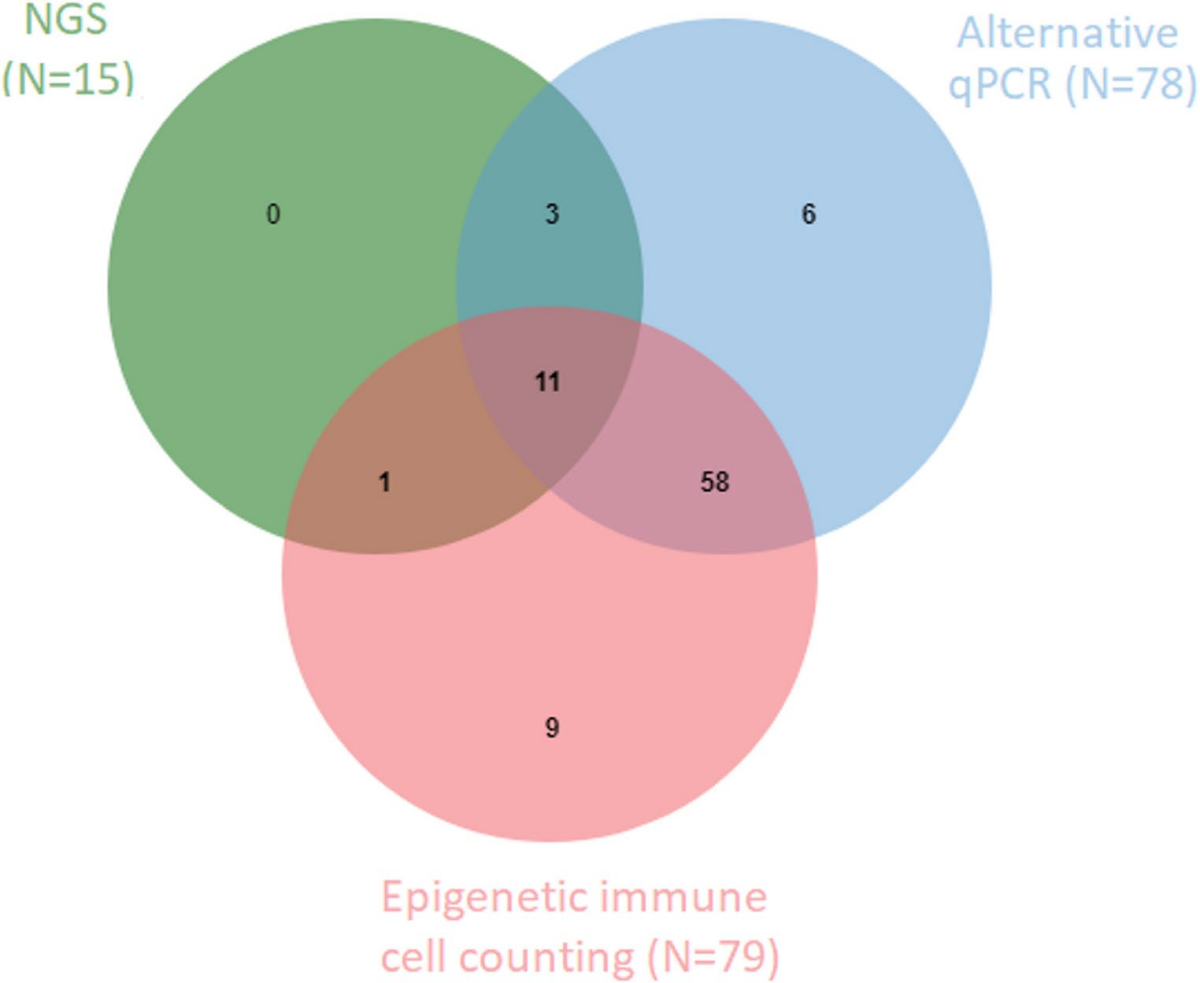
Venn-diagram of samples of newborns with low KRECS who that were included in all three second-tier tests (N=94) and would have been referred after initial KREC analysis based on the three different second tier test options; next generation sequencing (NGS), epigenetic immune cell counting and analysis with a second qPCR with different KREC primers

In total 94 samples were analyzed with all second tier test options. Figure 5 shows the samples of newborns who would have been referred based on either one of the three second tier test options: 15 newborns would have been referred in case NGS was used as second tier, 78 in case of an alternative qPCR multiplex assay and 79 newborns would have been referred if epigenetic immune cell counting was used as a second tier. Out of the 94 newborns with low KRECs, 11 newborns would have been referred irrespective of which second tier assay was used. All samples with potential variants shown in Table 2 had either low KREC levels measured with a second qPCR with different KREC primers or low relative epi-B cell counts measured with epigenetic immune cell counting. The exact KREC levels and relative B-cell counts of the XLA patients (positive controls) and the samples in which a potential variant was found are depicted in Table S2.

## Discussion

Early detection and timely treatment of XLA and other forms of agammaglobulinemia might result in less recurrent, severe infections and secondary complications, suggesting a role for detection via NBS. However, as NBS for B-lymphocyte deficiencies is based on the detection of the indirect and therefore aspecific marker KREC, screening is associated with a high number of secondary findings and a high referral rate. Our study is the first to evaluate multiple second tier test options after initial KREC measurement to enhance the positive predictive value of NBS for XLA and other forms of agammaglobulinemia.

A small reduction in the number of potential referrals was observed when using an alternative qPCR multiplex assay (94/110 newborns would have been referred). Correlation between the KREC levels measured with both commercially available assays was strong (Pearson r 0.83), suggesting that using an alternative qPCR multiplex assay might not be the most suitable second tier option but would be a potential alternative as a first tier. However, as some of the samples with KREC levels above the cut-off value of the manufacturer (PerkinElmer/Revvity) did have low epigenetic immune cell counts (*N* = 6) or variants in the B-cell deficiency panel as well as low epigenetic immune cell counts (*N* = 3), the exact test characteristics including sensitivity and specificity should be studied in a large scale pilot study. A more successful strategy might be to evaluate whether the cut-off value of either one of the assays can be lowered as the referral rate will be highly depending on the chosen cut-off. Although PCR with different primers might not result in a significant lower referral rate, this option does provide rapid availability of results with a feasible assay for any screening laboratory at relatively low costs [36].

Measuring relative cell counts in DBS with epigenetic immune cell counting is a promising technique in detection of IEIs shortly after birth [43]. In addition to XLA and other forms of agammaglobulinemia, epigenetic immune cell counting could be involved in early diagnosis of many IEIs that might benefit from early diagnosis and intervention if a suitable NBS test was available [44, 45]. Automating the protocol would be preferred to increase the throughput time for higher workloads. In our study, 86 out of 103 newborns would have been referred if epigenetic immune cell counting was applied as a second tier test. These results indicate that relative B-cell counts were low for most newborns with low KREC levels, suggesting a potential role for genetic testing as a second tier test to lower the number of referrals.

Many screening laboratories mainly use biochemical markers in their NBS program [46]. Recent studies however showed that the combination of biochemical marker with genetic analysis increases the positive predictive value and reduces the number of false-positives [47, 48]. Nowadays, more and more countries are exploring NGS technologies in NBS programs [49–51]. NGS with targeted gene panels will accelerate a final molecular diagnosis of affected newborns while providing useful information for management and follow-up. Targeted NGS has a rapid turnaround time, but is associated with relatively high analysis and equipment costs and a cost-effectiveness analysis should be performed prior to implementation [52].

In our study, targeted NGS seems a promising second tier test after KREC-analysis with the objective to reduce the number of (false-positive) referrals. In total, 16 children out of 103 newborns with low KRECs would be referred to a paediatric-immunologist for further diagnostics. It is important to note that deep-intronic, copy-number and structural variants can be missed with exon-based NGS and (likely) pathogenic variants could still be present in genes not included in the B-cell deficiency panel. A “safety net” construction should be in place referring newborns with the lowest KREC numbers in which no (likely) pathogenic variants were identified for follow-up immunophenotyping. Prior to implementation of NGS, evaluation of other sequence platforms and performance is required. One limitation of our study is that we did not include any healthy controls to help detecting sequence artefact more accurately. In addition, automated variant interpretation methods should be further explored as manual classification is time-consuming and more prone to inter-observer differences. When using targeted NGS, genes included in the panels need to be constantly updated and functional validation assays are of great importance to prove pathogenicity of novel variants [51].

Even though more and more countries are implementing NGS in NBS programs as second tier test, the use of NGS and other genetic tests as a first tier test in population screening remains disputed [53, 54]. Sequencing without any biological or phenotypic markers as a first tier remains challenging due to missing links between disease pathogenesis and gene expression and the inability to distinguish underlying pathogenic variants from the high number of benign genomic variations. Overall, rapid, high-throughput NGS analysis using targeted gene panels as a second tier after KREC analysis is a very promising technique to increase diagnostic precision and positive predictive value of the NBS program. However, prior to implementation, other aspects such as feasibility for screening laboratories, cost-effectiveness, ethical, legal and social implications should be evaluated in a broader perspective.

## Conclusions

In conclusion, all second tier tests have the potential to reduce the number of (false-positive) referrals and increase the positive predictive value of NBS for XLA and other forms of agammaglobulinemia. A second qPCR with different primers and epigenetic immune cell counting both led to a small reduction of the referral rate. Targeted NGS seemed to be the most promising second tier test, facilitating and accelerating molecular diagnoses of affected newborns. These findings will help NBS programs across the world in moving forward towards implementation of NBS for XLA and other forms of agammaglobulinemia.

## Supplementary Information

Below is the link to the electronic supplementary material.

ESM1 (19.7 KB)

ESM2 (94.2 KB)

## Data Availability

Data is provided within the manuscript or supplementary information files. The raw datasets generated during and/or analysed during the current study are available from the corresponding author on reasonable request.
