# Supplementary material for: Next-generation Sequencing and Other Second Tier Tests in Newborn Screening for (X-linked) Agammaglobulinemia
Source: J Clin Immunol. 2025 Nov 8;45(1):154. doi: 10.1007/s10875-025-01927-6 (PMC12594685; doi:10.1007/s10875-025-01927-6)

**Supplemental data**

**Table S1. Scoring system suspected sequence artefacts**

| **Characteristics** | **Score** |
| --- | --- |
| Homopolymer stretch ≥ 4 bases | +4 |
| Stretch ≥ 2 and <4 bases | +1 |
| Count*  1  2  3  ≥ 4 | +0  +1  +2  +3 |
| Only in forward strand or in reverse strand | +3 |
| In < 5% of forward strands or in reverse strands | +1 |
| Alternate allele ratio <0.25 | +2 |
| Phred Quality Score  <17  17-25  25-30  30-35 | +2  +1  +0.5  +0.25 |
| Coverage  <10  10-15  15-20  20-30 | +2  +1.5  +1  +0.5 |
| Described in population databases | -1 |

* Count: number of samples in which the variant was detected

**Table S2. KREC copies and relative B-cell counts of 4 XLA patients and 16 low KREC samples in which a potential (likely) pathogenic or heterozygous compound variant was identified.**

| **Sample ID** | **Gene and type of variant** | **KREC (ImmoIVD)**  **copies/3.2 mm punch** | **KREC (PerkinElmer/**  **Revvity) copies/10^5^ cells** | **Epi-B-cell counts (Epimune) %** |
| --- | --- | --- | --- | --- |
| XLA patient 1 | *BTK* Pathogenic | 1.44 | 0.0 | 0.08 |
| XLA patient 2 | *BTK* Pathogenic | 2.16 | 54.71 | 0.16 |
| XLA patient 3 | *BTK* Pathogenic | 0.74 | 0.0 | 0.1 |
| XLA patient 4 | *BTK* Pathogenic | 0.11 | *Not measured* | 0.05 |
| LOW KREC 77 | *BTK* Pathogenic | 5.10 | 621.97 | 0.34 |
| LOW KREC 7 | *CARD11* (Likely) pathogenic | 6.55 | 530.60 | 0.61 |
| LOW KREC 24 | *TOP2B* (Likely) pathogenic | 5.90 | 67.25 | 0.47 |
| LOW KREC 95 | *TOP2B* (Likely) pathogenic | 5.19 | 589.41 | 0.16 |
| LOW KREC 48 | *IRF2BP2* (Likely) pathogenic | 0.84 | 73.59 | 2.20 |
| LOW KREC 50 | *IRF2BP2* (Likely) pathogenic | 0.80 | 0.0 | 1.50 |
| LOW KREC 63 | *IRF2BP2* (Likely) pathogenic | 4.07 | 0.0 | 0.92 |
| LOW KREC 65 | *NFKB1* (Likely) pathogenic | 2.38 | 74.51 | 2.67 |
| LOW KREC 67 | *TCF3* (Likely) pathogenic | 0.62 | 0.0 | 0.38 |
| LOW KREC 73 | *TOP2B* (Likely) pathogenic | 2.53 | 449.91 | 0.40 |
| LOW KREC 76 | *SAMD9L* (Likely) pathogenic | 5.05 | 279.45 | 0.34 |
| LOW KREC 85 | *TCF3* (Likely) pathogenic | 4.66 | 189.28 | 0.26 |
| LOW KREC 91 | *CARD11* (Likely) pathogenic | 3.08 | 0.0 | 0.21 |
| LOW KREC 101 | *PGM3* (Likely) pathogenic  *PGM3* VUS  Potential compound heterozygous | 1.72 | 0.0 | 0.10 |
| LOW KREC 104 | *NFKB2* (Likely) pathogenic | 5.41 | *Not measured* | 0.05 |
| LOW KREC 74 | *FAT4* VUS (2 different variants)  Potential compound heterozygous | 3.29 | 193.21 | 0.36 |

**Figure S1. Distribution of KREC levels (copies/3.2 mm punch) in the population (N=110,491)**

**
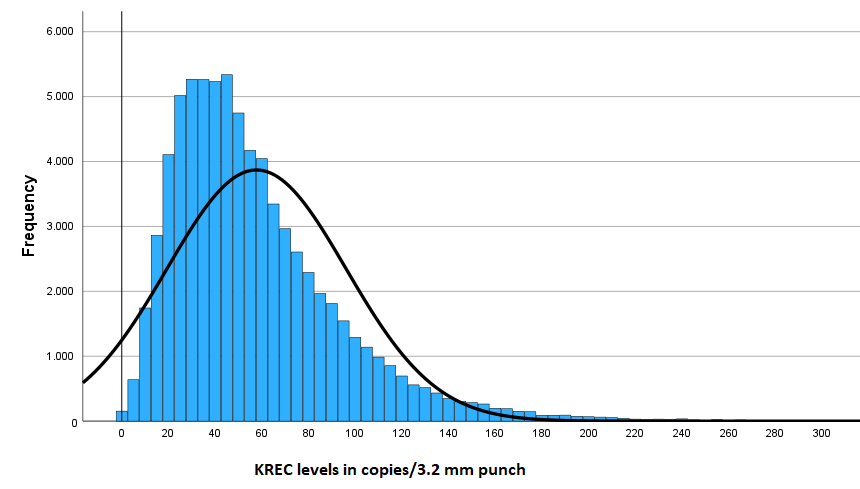
**

**Figure S2**. Genes in which variants were identified with NGS.


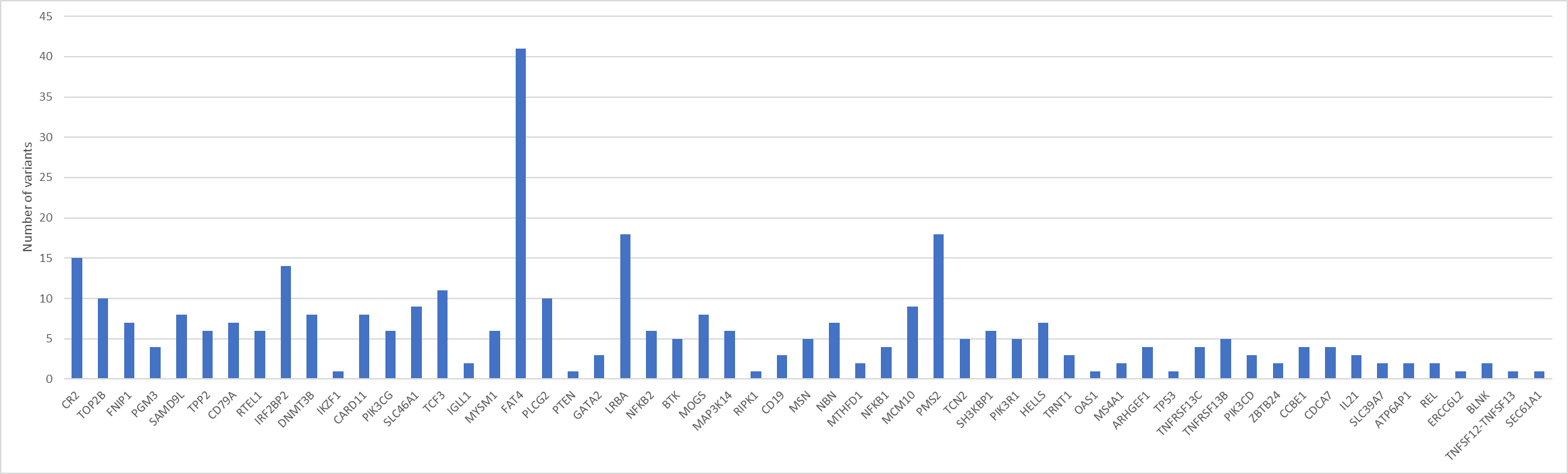

Supplement: Supplementary file 2 — ESM2 (94.2 KB) [file 10875_2025_1927_MOESM2_ESM.docx]
